# Sequential Infiltration Synthesis of Silicon Dioxide in Polymers with Ester Groups—Insight from In Situ Infrared Spectroscopy

**DOI:** 10.1021/acs.jpcc.3c07571

**Published:** 2024-04-03

**Authors:** Mahua Biswas, Vepa Rozyyev, Anil U. Mane, Amelia Korveziroska, Uttam Manna, Jeffrey W. Elam

**Affiliations:** † Department of Physics, Illinois State University, Normal, Illinois 61704, United States; ‡ Applied Materials Division, Argonne National Laboratory, Chicago, Illinois 60637, United States; § Advanced Materials for Energy-Water Systems (AMEWS) Energy Frontier Research Center, Argonne National Laboratory, 9700 South Cass Avenue, Lemont, Illinois 60439, United States; ∥ Pritzker School of Molecular Engineering, The University of Chicago, Chicago, Illinois 60637, United States

## Abstract

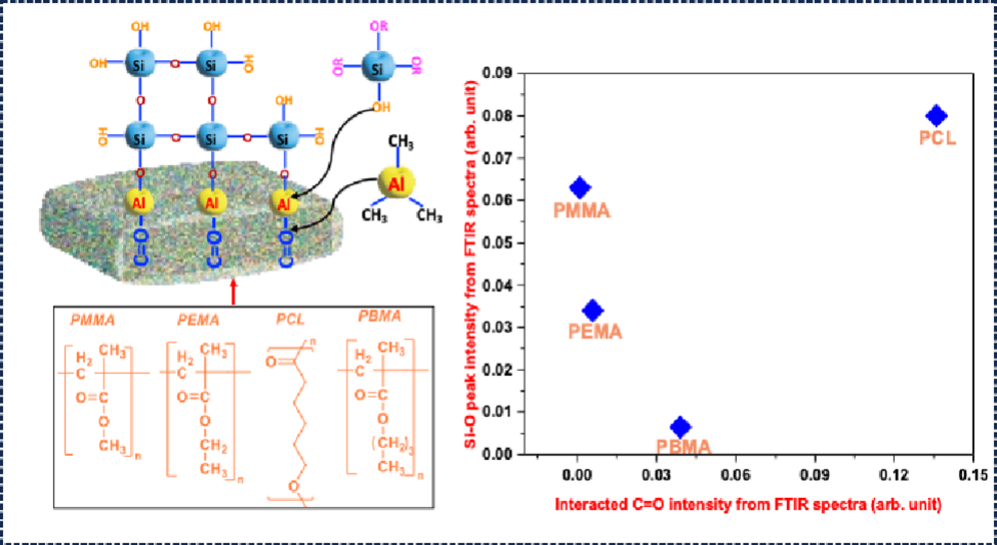

New strategies to synthesize nanometer-scale silicon dioxide (SiO_2_) patterns have drawn much attention in applications such as microelectronic and optoelectronic devices, membranes, and sensors, as we are approaching device dimensions shrinking below 10 nm. In this regard, sequential infiltration synthesis (SIS), a two-step gas-phase molecular assembly process that enables localized inorganic material growth in the targeted reactive domains of polymers, is an attractive process. In this work, we performed in situ Fourier transform infrared spectroscopy (FTIR) measurements during SiO_2_ SIS to investigate the reaction mechanism of trimethylaluminum (TMA) and tri(tert-pentoxy) silanol (TPS) precursors with polymers having ester functional groups (poly(methyl methacrylate) (PMMA), poly(ethyl methacrylate) (PEMA), polycaprolactone (PCL), and poly(t-butyl methacrylate) (PBMA)), for the purpose of growing patterned nanomaterials. The FTIR results show that for PMMA and PEMA, a lower percentage of functional groups participated in the reactions and formed weak and unstable complexes. In contrast, almost all functional groups in PCL and PBMA participated in the reactions and showed stable and irreversible interactions with TMA. We discovered that the amount of SiO_2_ formed is not directly correlated with the number of interacting functional groups. These insights into the SiO_2_ SIS mechanism will enable nanopatterning of SiO_2_ for low-dimensional applications.

## 1 Introduction

Silicon dioxide (SiO_2_) has been used extensively as a dielectric material in the microelectronics industry since the 1960s. The outstanding properties of SiO_2_^1,2^ including excellent dielectric breakdown strength (>10^9^ V/m), high resistivity (>10^14^ Ω·cm), large band gap (∼9 eV),^3^ and compatibility with current silicon-based devices, make it ideal for many applications in microelectronics and optoelectronics. In addition, with the rapid development of nanoscale devices and technologies, nanoscale patterns of SiO_2_ have been used for various applications, such as biosensing,^4^ nanometer scale membranes,^5^ and information technology,^6^ to fulfill ever increasing demands of nanoscale devices with regards to dimensionality. SiO_2_ nanopatterning is typically performed by using optical lithography or electron beam lithography. However, the feature size in optical lithography is limited by the wavelength of light, and electron beam lithography has limited throughput due to the serial nature of the patterning. In addition, both methods rely on polymeric resist masks that suffer from pattern collapse and limited etch resistance when patterning small features.^7^ Consequently, new methods of nanofabrication are being considered to overcome the limitations of established nanopatterning techniques.^8−12^

A growth process for fabricating well-ordered SiO_2_ nanostructures with controllable dimensions and material morphology would be very attractive for emerging devices. In this regard, sequential infiltration synthesis (SIS),^13−15^ which is used for block copolymer (BCP)-assisted inorganic nanopatterning^12,16−24^ by infusing polymer materials with inorganic compounds, could be used to fabricate well-ordered SiO_2_ nanostructures. The BCP-assisted templating of SiO_2_ nanostructures has been explored previously using sol–gel processing with silicon tetrachloride (SiCl_4_) and H_2_O.^25,26^ However, this method is difficult to control due to homogeneous reactions and surface-limited reaction processes. In contrast, SIS involves the self-limiting reaction of precursor vapors by sequential exposure of two precursors participating in a binary reaction. SIS has been extensively explored for materials such as Al_2_O_3_, TiO_2_, ZnO, and W.^12,21,24,27−36^ Some factors that make SIS very attractive are: (i) it can overcome the pattern resolution limitation of conventional lithography^8^ by selecting nm-scale polymer nanostructures as templates, (ii) the patterning template can be formed easily and at low cost using self-assembled polymers such as BCPs or nanostructured polymers, (iii) the selective infiltration of inorganic material in SIS eliminates patterning steps of preparing the template, and (iv) the polymer template is easily removable at the end and compatible with current semiconductor fabrication processing which uses polymer resists for nanopatterning. The above-mentioned effectiveness of SIS for advanced nanopattering is a driving force toward exploring more SIS materials. Hence, nanopatterning SiO_2_ using SIS with polymer templates will be an attractive route for fabricating well-ordered SiO_2_ nanostructures for existing and emerging applications. However, a detailed understanding of the SiO_2_ SIS reactions in different polymer materials is required to design effective SiO_2_ nanopatterning processes.

**Figure 1:**
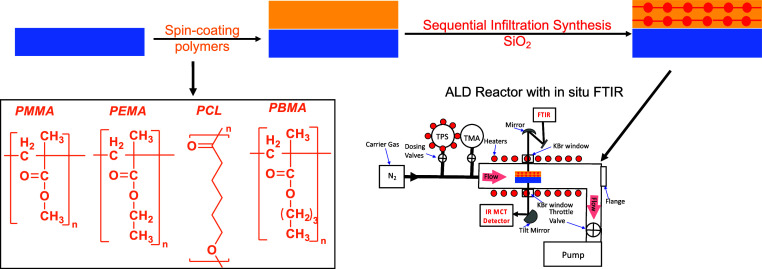
Schematic illustration of SIS SiO_2_ in ester-based polymers and an in situ FTIR study setup illustrated in this work.

In this work, we performed in situ FTIR measurements following individual SIS precursor exposures (half-cycles) and after full cycles of SiO_2_ SIS inside a range of ester-based polymers to gain insight into the precursor-polymer interactions and the nature of the hybrid molecular assembly for the purpose of utilizing the process for SiO_2_ nanopatterning. More specifically, we explored SiO_2_ SIS in four homopolymer thin films: poly(methyl methacrylate) (PMMA), poly(ethyl methacrylate) (PEMA), polycaprolactone (PCL), and poly(t-butyl methacrylate) (PBMA) (Figure 1). SiO_2_ SIS has been demonstrated previously using polystyrene-*block*-poly(methyl methacrylate) (PS-*b*-PMMA) as a template but there is very little understanding of the SIS process in this material.^17^ Understanding the chemical interactions between the infiltrated polymer and the SIS precursors is crucial for process design and optimization, improving the quality of the nanopatterns, such as reducing defects and line edge roughness, and identifying new polymers and precursors that will broaden the nanopatterning capabilities and improve the overall deposition. In this regard, FTIR is a powerful technique and provides an opportunity to study the interactions between chemical species by measuring the absorption peaks corresponding to distinct vibrational frequencies of the precursor, polymer, and potential complexes or reaction products between them to identify the functional groups and species involved in the interactions.^37−39^ Therefore, the results from this work will be instrumental in establishing SiO_2_ SIS by providing insight into the process chemistry.

The four homopolymer thin films mentioned above were selected to investigate the interactions of the functional groups bonded in these polymers with the same two precursors. Trimethylaluminum (TMA, Al(CH_3_)_3_) and tri(tert-pentoxy) silanol (TPS, (CH_3_CH_2_C(CH_3_)_2_O)_3_SiOH) were selected as the SiO_2_ SIS precursors. A variety of thermal Atomic layer deposition (ALD) processes for SiO_2_ have been evaluated previously, such as SiCl_4_ and H_2_O,^40^ Si(NCO)_4_ and H_2_O,^41^ (H_3_CO)Si(NCO)_3_ and H_2_O_2_,^42^ and Si(NCO)_4_ and N(C_2_H_5_)_3_.^43^ However, these processes require long reactant exposure times that are likely not viable for manufacturing. Alternative SiO_2_ ALD processes have been developed using ozone as the second precursor,^44^ and O_2_/N_2_ plasma along with Si(N(CH_3_)_2_)_3_ and Si(N(CH_3_)_2_)_4_ for plasma-assisted ALD (PEALD) to grow SiO_2_ films.^45^ However, O_2_/N_2_ plasmas are likely to degrade or etch the organic polymer templates and are therefore unsuitable for SIS. Although O_3_ has been used successfully for Al_2_O_3_ SIS in PS-*b*-PMMA,^46^ O_3_ is a powerful oxidant and may damage some polymer templates.^47^ In addition to these conventional ALD processes, alternative SiO_2_ deposition methods have been developed using silanols with catalysts such as aluminum (Al), hafnium (Hf), and zirconium (Zr) which show rapid SiO_2_ growth while maintaining the self-limiting nature of ALD.^17,48−52^ The precursors used for these types of catalyzed ALD include TPS and tris(tert-butoxy)silanol (TBS). Considering the principle and observations from these studies of faster and localized SiO_2_ growth, we selected TMA and TPS for this work to study the SiO_2_ SIS process in different polymers. To the best of our knowledge, the interaction mechanism of TPS with TMA-treated polymers is reported for the first time in this work.

Our results showed different degrees of interaction for these polymers with the SiO_2_ SIS precursors even though the polymers share the same ester functional groups consisting of carbonyl (C=O) and C–O–R species (Figure 1). PMMA and PEMA both showed similar interactions with TMA, where we observed lower functional group participation and weak and unstable complex formation, whereas for PCL and PBMA, almost all functional groups participated in the interaction and showed stable and irreversible interactions with TMA. The Si–O formation after TPS exposure was evaluated from the Si–O peak intensity in the FTIR spectra and we found that it was not directly correlated with the number of polymer functional group interactions. The PCL polymer showed maximum Si–O formation, followed by PMMA, PEMA, and PBMA. Even though all of the functional groups of PBMA interacted after TMA exposure similarly to PCL, the intensity of the Si–O peaks was less intense, indicating possible intermediate complex formation due to incomplete silanol transformation to siloxane. The FTIR data provide a detailed understanding of the polymers that can be useful for infiltration and the amount and nature of the deposition that can be expected for these polymers using TMA and TPS from the changes in the vibrational peak intensity in the spectra. The results of this study will provide a guideline for SiO_2_ SIS nanopatterning and open up possibilities for exploring SiO_2_ nanopatterning with these polymers. The results will also help in the exploration of new precursors for the SIS process.

## 2 Experiments

In this work, polymer thin films of four different homopolymers were spin-cast on double-side polished Si(100) wafers (University Wafers) with native oxide intact for the SIS experiments. The Si(100) substrates were undoped and had a high resistivity of >10,000 Ω·cm and a thickness of 525 μm so as to be optically transparent in the IR wavelength range for our measurements. The four polymers used in this work are PMMA (*M*_w_ = 467 K), PEMA (*M*_w_ = 377 K), PCL (*M*_w_ = 96.5K), and PBMA (*M*_w_ = 184.8K) and were purchased from Scientific Polymer Products, Inc. The Si substrates were cleaned with isopropyl alcohol (IPA) in the spin coater by dropping IPA on the Si substrate and spinning for 45 s at 4500 rpm prior to applying the polymer solution. Polymer films were deposited on the cleaned Si substrates by spin coating at 4500 rpm for 45 s from 3 weight% solutions of PMMA in acetone, PEMA in acetone, PCL in toluene, and PBMA in acetone. All polymer films were annealed at 120 °C for 180 s on a hot plate to evaporate the solvents. The thickness of the polymer films was measured using an Ellipsometer (J. A. Woollam Co. Alpha-SE).

SiO_2_ SIS experiments were performed by alternating exposures of TMA (Aldrich, 97%) and TPS (Aldrich 99.99%) inside a custom, hot-walled, viscous flow ALD/SIS reactor that has been described previously.^47,53^ (Caution: TMA is a pyrophoric liquid.) As TMA has a high vapor pressure, this precursor was kept at room temperature, while the lower vapor pressure TPS was loaded in a stainless steel bubbler and heated to 120 °C. The sample stage and reactor walls were maintained at 125 °C during the experiments to avoid condensation of the TPS precursor on the reactor wall during precursor exposures. The in situ FTIR measurements were performed in transmission mode using a Nicolet 6700 FTIR spectrometer (Thermo Scientific) interfaced to a custom ALD/SIS reactor that has been described previously and in Figure 1.^47,53,54^ SIS was performed in static mode wherein the reactor was first evacuated to ∼50 mTorr, and the exhaust valve to the vacuum pump was closed prior to introducing the precursor vapors. During the precursor exposures, gate valves were closed to isolate the IR transparent windows from the ALD/SIS reactor to prevent growth on the windows. The dosing valve to the first precursor (TMA) was opened for 10 s. After the dosing valve was closed, the polymer film was subjected to a 90 s static exposure. Following this dose/exposure protocol, the exhaust valve was opened, and the chamber was purged with ultrahigh purity (99.999%) N_2_ gas at 350 sccm for a purge period of 60 s to complete the first half cycle of the SiO_2_ SIS reaction. The same procedure was followed for the TPS precursor to complete one cycle of the SiO_2_ SIS reaction. During the 10 s TPS dose, 15 sccm of ultrahigh-purity N_2_ gas flowed through the TPS bubbler. FTIR spectra were collected after each precursor exposure following a 35 s delay to ensure adequate purging of the SIS precursors from the chamber to avoid deposition on the FTIR windows. Each FTIR spectrum was then collected for 320 s with an average of 256 scans recorded at a resolution of 4 cm^–1^.

## 3 Results

To understand the reaction mechanism of this localized SiO_2_ deposition process inside different polymers, in situ FTIR spectra were recorded for each as-grown polymer and after the TMA and TPS precursor exposures during the SIS experiments to observe changes in the chemical structures. The polymer spectrum and the difference spectra are shown and described separately for each of the four polymers. As mentioned above, the polymer films were deposited on Si substrates with native SiO_2_, and a background spectrum from a bare substrate was recorded prior to each polymer experiment. This background spectrum was subtracted from all of the subsequent FTIR spectra recorded from the polymer films during data collection.

**Figure 2:**
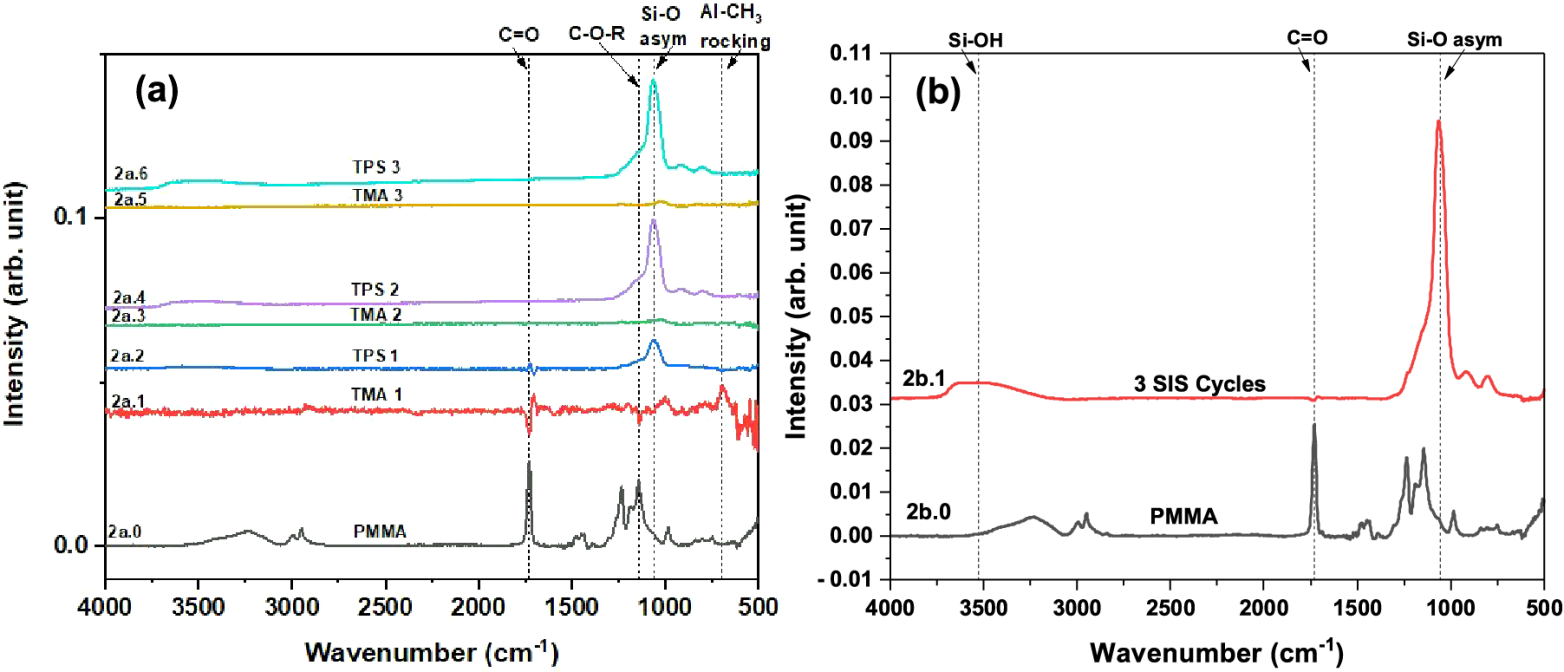
FTIR absorbance spectra recorded following exposure of TMA and TPS to PMMA. (a) As grown PMMA spectrum (2a.0) and difference spectra after each precursor exposure during three SIS cycles, and (b) PMMA as grown (2b.0) and difference spectrum (2b.1) after three complete SIS cycles. Note, spectrum 2a.1 is 10× magnified to observe all the changes clearly, and spectrum 2a.2 is 10× magnified only in the 1770 cm^–1^–1685 cm^–1^ wavelength (C=O) region to observe the changes clearly in that region.

**Figure 3:**
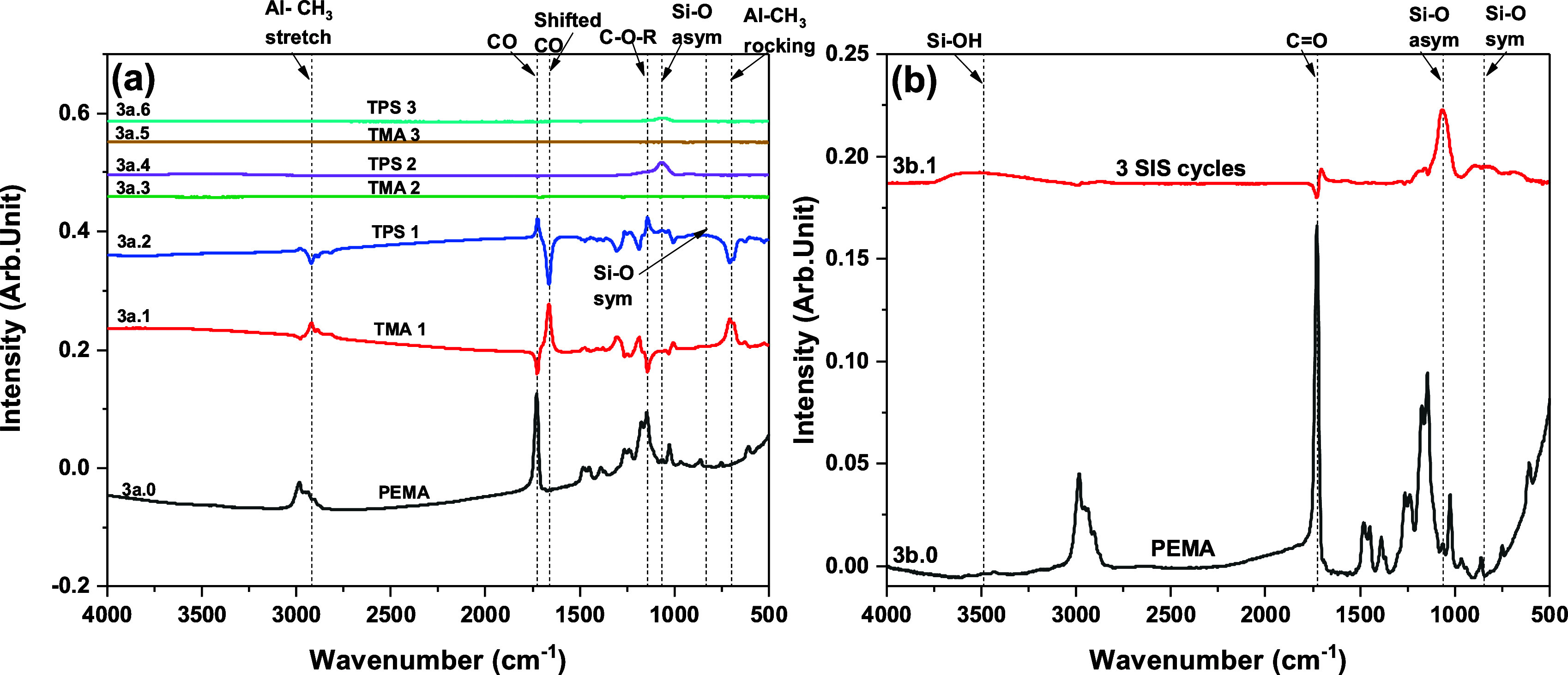
FTIR absorbance spectra recorded following exposure of TMA and TPS to PEMA. (a) As grown PEMA spectrum (3a.0) and difference spectra after each precursor exposure during three SIS cycles, and (b) PEMA as grown (3b.0) and difference spectrum (3b.1) after three complete SIS cycles.

**Figure 4:**
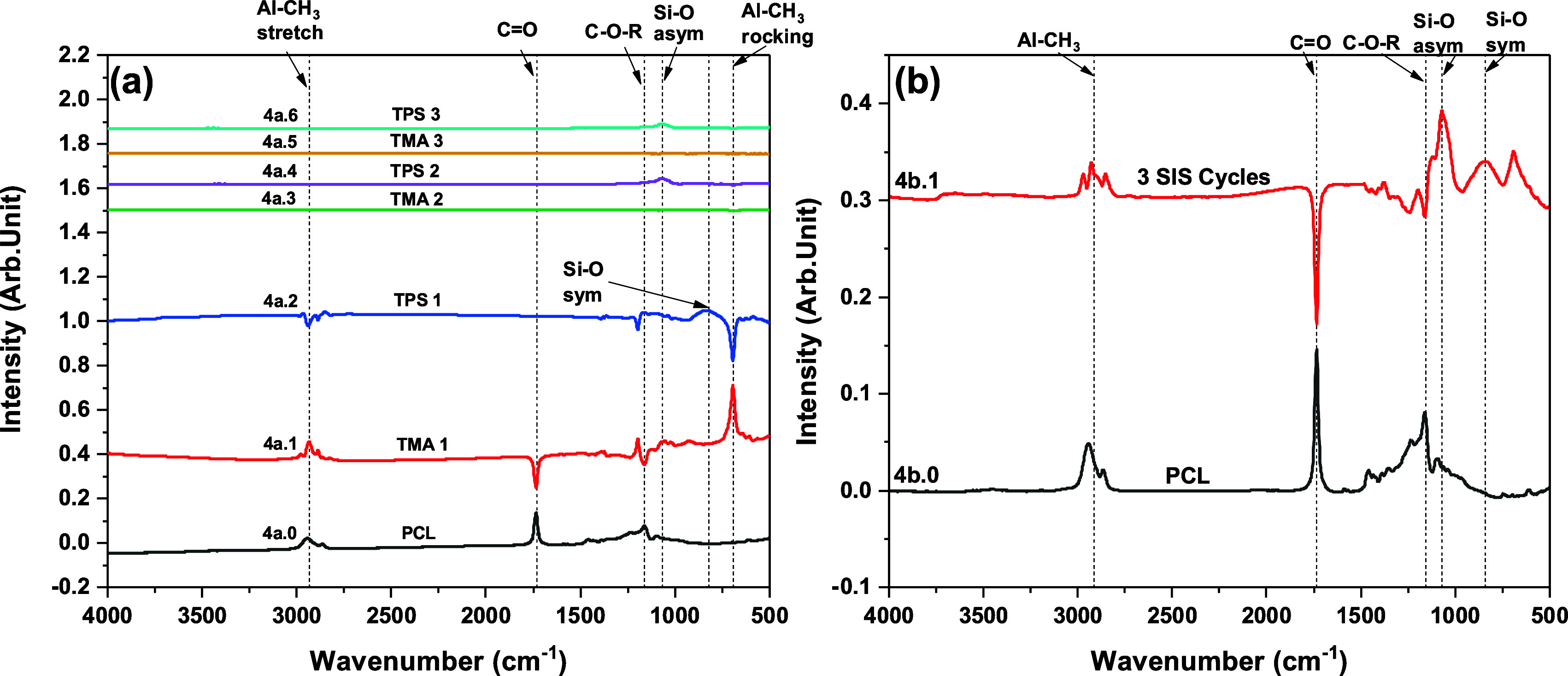
FTIR absorbance spectra recorded following exposure of TMA and TPS to PCL. (a) As grown PCL spectrum (4a.0) and difference spectra after each precursor exposure during three SIS cycles, and (b) PCL as grown (4b.0) and difference spectrum (4b.1) after three complete SIS cycles.

**Figure 5:**
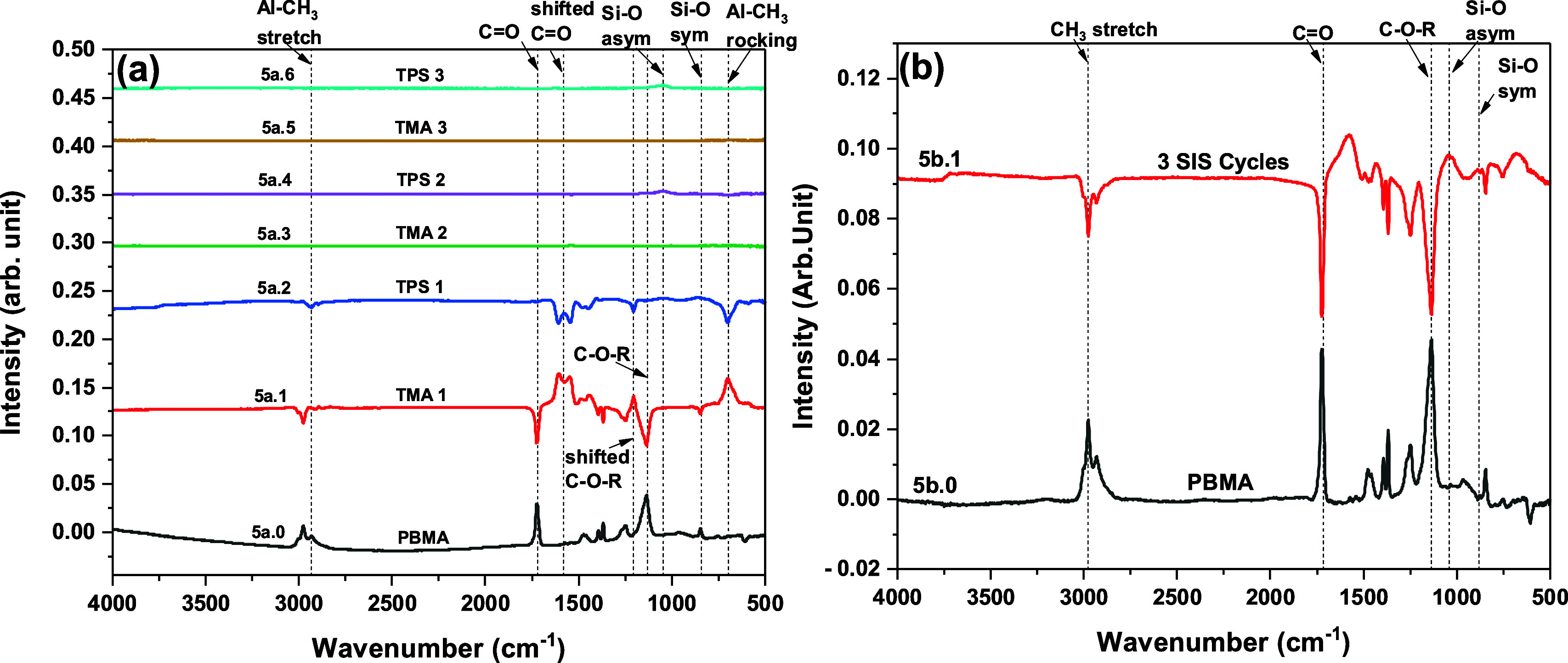
FTIR absorbance spectra recorded following exposure of TMA and TPS to PBMA. (a) As grown PBMA spectrum (5a.0) and difference spectra after each precursor exposure during three SIS cycles, and (b) PBMA as grown (5b.0) and difference spectrum (5b.1) after three complete SIS cycles.

We have plotted the difference spectra to highlight the chemical changes after each SIS half cycle. The difference spectra are obtained by subtracting the previous spectrum from the current spectrum. Consequently, positive features in the difference spectrum signify newly formed species, and negative features indicate consumed species. However, for Figures 2–5 spectra 2b.1, 3b.1, 4b.1, and 5b.1, the difference spectrum after three SIS cycles was obtained by subtracting the pristine PMMA spectrum from the spectrum recorded after three complete SIS cycles. As a result, Figures 2–5 spectra 2b.1, 3b.1, 4b.1, and 5b.1 show the cumulative effect of the three SIS SiO_2_ cycles on the FTIR spectrum.

### 3.1 Poly(methyl methacrylate) (PMMA)

The interaction of TMA with PMMA during Al_2_O_3_ SIS and ALD has been studied extensively by our group and others.^37,39,54−56^ Here, we show the interactions of ∼163 nm thick PMMA with TMA followed by TPS during three SIS cycles.

Figure 2a,b shows the spectrum of the pristine PMMA film (spectra 2a.0 and 2b.0) and the difference spectra (spectra 2a.1–2a.6) obtained following each half cycle of the SiO_2_ SIS. Spectrum 2b.1 in Figure 2b shows the net change in the polymer film after three complete SIS cycles. The difference spectra in Figure 2a are also shown in Figure S1 to exhibit some of the smaller spectral features more clearly. It is important to note that the nucleation of the polymer sites with the precursors during the first cycle of SIS is crucial, as it determines the nature of infiltration of the material within the polymer.

As mentioned above, we have described the in situ FTIR measurements of TMA exposure on PMMA in our previous works and discussed the chemical interactions between PMMA and TMA in detail.^54,55^ Briefly, the FTIR spectrum of clean PMMA (spectrum 2a.0) shows the following peaks: C=O stretching at 1729 cm^–1^, C–O–R stretching at 1236 and 1145 cm^–1^, symmetric CH stretching of CH_3_ at 2954 cm^–1^, CH stretching of CH_2_ at 2994 cm^–1^, and CH bending of CH_3_ and CH_2_ at 1438 and 1478 cm^–1^.^38,57−59^ Following TMA exposure, the difference spectra 2a.1 and S1a.1 show two negative features (peaks below the baseline) at 1729 and 1145 cm^–1^ due to the consumption of C=O and C–O–R groups, respectively. The positive features (peaks above the baseline) in spectra 2a.1 and S1a.1 are due to Al-CH_3_ rocking at ∼700 cm^–1^, and red shifting of the 1729 cm^–1^ C=O peak at 1706 cm^–1^.^37,47,54,60^ The red-shifted 1726 cm^–1^ C=O peak is due to the formation of a weakly bound C=O•••Al(CH_3_)_3_ complex.^61^ In Figure 2, spectrum 2a.2 and and Figure S1, spectrum S1a.2, the difference spectrum after TPS exposure on the TMA treated PMMA is shown. After the TPS exposure, which completes the first SiO_2_ SIS cycle, we see the appearance of an intense broad peak in the ∼950–1300 cm^–1^ region due to Si–O asymmetric stretching indicating the formation of a siloxane chain.^62−64^ The negative peak at ∼700 cm^–1^ in spectrum 2a.2/S1a.2 results from the removal of TMA methyl groups signaling Al-bound Si–O formation by replacement of the methyl groups of Al−CH_3_ with the O–Si species. The broad peak ∼3000–3750 cm^–1^ in spectrum 2a.2/S1a.2 is attributed to Si–OH stretching from unreacted silanol groups (Si–OH) and will be discussed in Section 4. We also see a nearly symmetric reversal of the C=O feature observed in spectra 2a.1 and S1a.1. Note that the partial release of C=O groups after the TPS exposure, as seen from spectrum 2a.2/S1a.2, is an indication of some Al–O–Si formation in the unbound form to PMMA; a similar observation which was discussed in our previous work and by McGuinness et al. for Al–O formation in the unbound form during Al_2_O_3_ growth in PMMA.^54,65^ Spectra 2a.3–2a.6 do not show further changes to the polymer functional groups of PMMA. We observe in spectra 2a.3 and 2a.5 the appearance of the Al-CH_3_ symmetric deformation mode at ∼1050 cm^–1^ after TMA exposure. Spectra 2a.4 and 2a.6 show the appearance of an intense and broad Si–O peak at ∼950–1300 cm^–1^ and a Si–OH peak at ∼3000–3750 cm^–1^. The absence of changes in the PMMA functional groups in spectra 2a.3 and 2a.5 indicates that a majority of the reaction and formation of Si–O is on existing SiO_2_ nuclei embedded in the PMMA during the second and third SiO_2_ SIS cycles instead of reaction with the polymer. It was also evident from the small broad peak in the 600–800 cm^–1^ region of spectra S1b.1 and S1c.1 (not visible in Figure S1a.1 due to the low intensity) that TMA reacts with Si–OH/Si–O–Si. Figure 2b spectrum 2b.1 shows the cumulative changes after three SiO_2_ SIS cycles and reveals the loss of C=O and C–O–R and intense Si–O and Si–OH formation consistent with the discussion above.

### 3.2 Poly(ethyl methacrylate) (PEMA)

The interactions of TMA and TPS with PEMA (Figure 2) show features similar to the SiO_2_ SIS on PMMA. The FTIR spectrum of the pristine ∼199 nm thick PEMA film (spectra 3a.0 and 3b.0) and the difference spectra (spectra 3a.1–3a.6) obtained during half cycles of the SiO_2_ SIS on PEMA are shown in Figure 3a. The difference spectra in Figure 3a are also shown in Figure S2 to more clearly exhibit some of the smaller spectral features.

Similar to PMMA, the FTIR spectrum of pristine PEMA (spectrum 3a.0) shows the following major peaks: C=O stretching at 1724 cm^–1^, C–O–R stretching at 1250 and 1140 cm^–1^, symmetric CH stretching of CH_3_ at 2942 cm^–1^, CH stretching of CH_2_ at 2988 cm^–1^, and CH bending of CH_3_ and CH_2_ at 1450 and 1483 cm^–1^.^38,57−59^ From the difference spectrum 3a.1/S2a.1 after TMA exposure, we see the interactions of the C=O and C–O–R from the negative peaks at 1724 cm^–1^ and 1140 cm–1, respectively, and the following positive peaks appear: Al-CH_3_ rocking at ∼700 cm^–1^, red shifting of the 1724 cm^–1^ C=O peak at 1661 cm^–1^, and blue shifting of the C–O–R peak at ∼1186 cm^–1^. We also observe a C–H stretching mode from Al−CH_3_ at ∼2920 cm^–1^ after TMA exposure in spectrum 3a.1/S2a.1.^37,60^ After the subsequent TPS exposure (spectrum 3a.2/S2a.2), we see a broad peak in the ∼950–750 cm^–1^ region, which can be identified as the Si–O symmetric stretching vibration and a small peak at ∼1063 cm^–1^ due to Si–O asymmetric stretching. In addition, a reversal happens for all the positive and negative peaks observed in spectrum 3a.1/S2a.1, including C=O, C–O–R, shifted C=O and C–O–R, and the Al−CH_3_ asymmetric and rocking modes.

The FTIR difference spectra for the second and third SiO_2_ SIS cycles are shown in spectra 3a.3–3a.6 and in more detail in Figures S2b,c. The second TMA exposure produces a weak negative peak in the C=O region (visible in Figure S2b), indicating some PEMA C=O interaction. After the second TPS precursor exposure, we observe from the difference spectrum 3a.4 an Si–O asymmetric stretching peak ∼1063 cm^–1^ and a small Si–O symmetric stretching peak centered ∼913 cm^–1^.^63,64,66^ We also observe a broad peak at ∼3000–3750 cm^–1^ attributed to Si–OH, which was not quite visible after the completion of the first SIS cycle. During the third SIS cycle, virtually no changes are seen after the TMA exposure (spectrum 3a.5). However, we observe the appearance of the Si–O asymmetric peak at ∼1063 cm^–1^ and a small Si–OH peak at ∼3500 cm^–1^ after the TPS exposure (spectrum 3a.6). There was no peak at ∼900 cm^–1^ due to Si–O symmetric vibration after the completion of the second and third SIS cycles. The cumulative changes after three complete SIS cycles (Figure 3b, spectrum 3b.1) show the interacted (negative) C=O and C–O–R peaks, the appearance of the (positive) red-shifted C=O peak, Si–O asymmetric and symmetric peaks centered ∼1063 and 850 cm^–1^, respectively, and the Si–OH peak in the ∼3000–3750 cm^–1^ region.

### 3.3 Polycaprolactone (PCL)

Our group recently reported an in situ FTIR study of Al_2_O_3_ SIS in PCL using TMA and H_2_O.^56^ We found significant interactions between the PCL polymer functional groups and TMA during the first cycle that were more extensive than those in previous SIS experiments with other polymers. Our in situ FTIR measurements of SiO_2_ SIS in ∼123 nm thick PCL are shown in Figure 4. The clean PCL polymer spectrum (spectra 4a.0 and 4b.0) shows the following peaks: C=O stretching at 1733 cm^–1^, C–O–R stretching at 1231 and 1162 cm^–1^, CH_2_ asymmetric stretching at ∼2942 cm^–1^, and CH_2_ symmetric stretching at ∼2860 cm^–1^.^38,67^ The difference spectrum 4a.1/S3a.1 in Figures 4a, S3 following TMA exposure reveals a near-complete removal of the C=O peak without formation of a red-shifted C=O feature. The absence of a red-shifted C=O feature in spectra 4a.1/S3a.1, unlike with the PMMA and PEMA polymer films, indicates that TMA forms a covalent bond to the C=O group in PCL rather than a physisorbed complex. Moreover, the magnitude of the C=O loss feature in spectrum 4a.1 is similar to that of the C=O feature in the original PCL film (spectrum 4a.0) suggesting that nearly all the C=O groups are consumed when TMA interacts with PCL. The C–O–R feature at 1162 cm^–1^ is also lost upon TMA reaction with PCL, but in this case, a blue-shifted positive peak appears at ∼1199 cm^–1^, which is similar to the PMMA-TMA and PEMA-TMA interactions. We also observe Al-CH_3_ stretching and rocking modes at 2938 and 700 cm^–1^, respectively, in spectra 4a.1/S3a.1.^38,60^

Spectrum 4a.2/S3a.2 is the difference spectrum following TPS exposure and shows that the positive peaks for the blue-shifted C–O–R and Al-CH_3_ stretching and rocking vibrations from spectrum 2a.2/S1a.2 are reversed, indicating that TPS reacts with the Al−CH_3_ and complexed C–O–R species. No changes are seen in the C=O feature in 4a.2/S3a.2, again signaling the complete and irreversible reaction of the PCL C=O group with TMA. The formation of Si–O species upon TPS exposure is indicated by a broad peak in the 950–750 cm^–1^ region attributed to the Si–O symmetric vibration, as seen for PEMA (mentioned in Section 3.2). We do not observe any broad and intense Si–O asymmetric peak after the first SIS cycle. In the subsequent two SiO_2_ SIS cycles (spectra 4a.3–4a.6), we do not observe any further changes in the C=O and C–O–R regions, indicating no further interactions with the polymer functional groups. We observe the Si–O asymmetric peak at ∼1073 cm^–1^ after TPS exposure (spectra 4a.4 and 4a.6) and a negative peak at ∼700 cm^–1^ (as shown in spectra S3b.2 and S3c.2)) indicating reaction of Al-CH_3_ from the previous cycle after TPS exposure. The cumulative changes after three complete SIS cycles are shown in spectrum 4b.1 and reveal the interacted (negative) C=O and C–O–R peaks and appearance of (positive) Si–O asymmetric, Si–O symmetric, and Si–OH peaks. We also see a positive peak ∼3000–2800 cm^–1^ region which can be due to unreacted Al-CH_3_ species.^62^

### 3.4 Poly(t-butyl methacrylate) (PBMA)

Figure 5 shows the FTIR absorbance spectra recorded following TMA and TPS exposures to ∼230 nm thick PBMA films. (Note: the thickness data fitting mean squared error in the ellipsometer was higher than the other three polymers in multiple trials.) The initial PBMA spectrum 5a.0/5b.0 predominantly shows C=O, C–O–R, CH_3_ stretching, and CH_2_ stretching peaks at 1720 cm^–1^, 1132 cm^–1^, 2974 cm^–1^, and 2934 cm^–1^, respectively.^68,69^ After the first TMA exposure (spectrum 5a.1/S4a.1), we see nearly complete consumption of the C=O and C–O–R peaks from PBMA (as seen from the peak intensity reversal). We also observe two red-shifted C=O peaks at 1606 and 1546 cm^–1^ and a blue-shifted C–O–R features at 1204 cm^–1^. The two peaks for red-shifted C=O may indicate the formation of weakly bound C=O···Al-(CH_3_)_3_ complexes with two different bond energies. Additionally, the Al-CH_3_ rocking mode is visible at 700 cm^–1^, and a small peak at 2938 cm^–1^ appears for C–H stretching in Al-CH_3_. The TPS exposure reverses the red-shifted C=O, blue-shifted C–O–R, and Al-CH_3_ rocking peaks, as shown in spectrum 5a.2/S4a.2. We see a small broad peak centered at ∼860 cm^–1^ due to the Si–O symmetric vibration, a peak at 1045 cm^–1^ due to the Si–O asymmetric vibration and another broad peak in the ∼3000–3750 cm^–1^ region for Si–OH. Note that we do not see the reappearance of the C=O and C–O–R species similar to PCL and unlike PMMA and PEMA, which indicates an irreversible reaction of the C=O and C–O–R groups of PBMA with TMA. In the difference spectra plotted after TMA and TPS exposures for subsequent SIS cycles (spectra 5a.3–5a.6), only a small Si–O peak after TPS exposure is visible at 1053 cm^–1^ due to the Si–O asymmetric vibration. The difference spectra after three SIS cycles (spectrum 5b.1) show the loss (negative peaks) of C=O, C–O–R, and C–H stretching and the appearance (positive peaks) of low-intensity Si–O asymmetric and Si–O symmetric peaks. The interactions of PBMA with TMA show a very similar trend as PCL (Section 3.3) with strong and irreversible polymer group interactions. However, the intensity of the Si–O peaks is smaller after TPS exposure in PBMA compared to PCL.

## 4 Discussion

Interestingly, the four polymers with the same types of functional groups at different positions within the corresponding molecule showed different degrees of interaction. The active functional groups which interacted strongly in the SIS reactions with the TMA and TPS are C=O and C–O–R for all four polymers PMMA, PEMA, PCL, and PBMA. The FTIR data presented in Figures 2–5 provide a deeper understanding of the SiO_2_ SIS process in these four different polymers and discussed below.

### 4.1 Growth Mechanism

**Figure 6:**
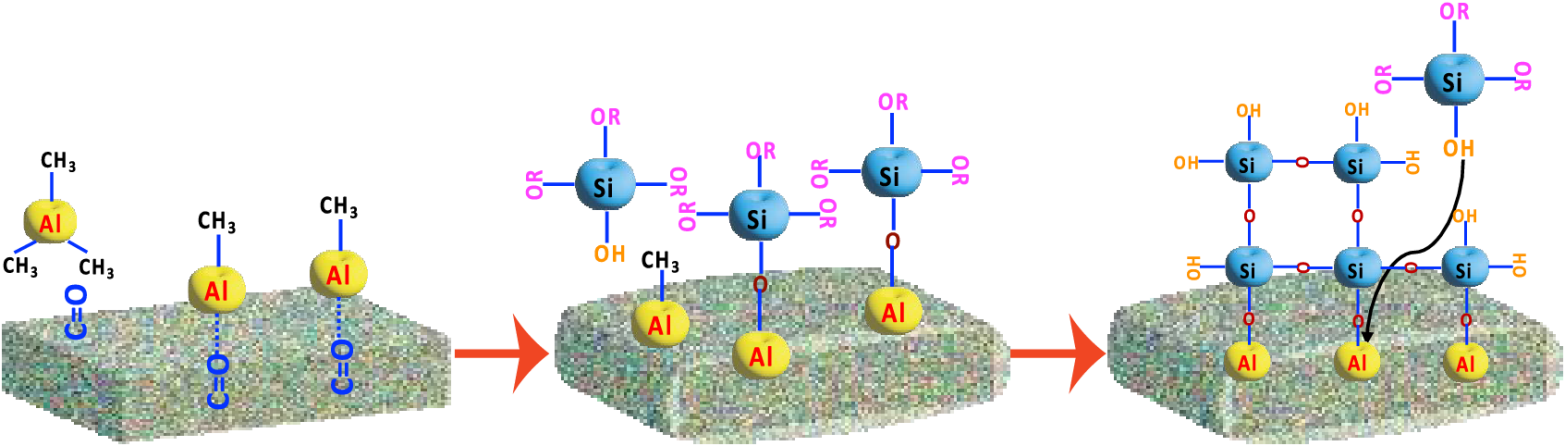
Block diagram of the proposed growth mechanism of Si–O materials inside polymers with C=O/C–O-R groups.

The reaction mechanism for the process is proposed as the polymer functional groups (C=O and C–O–R) interact/react with TMA in the first step of the binary reaction. In the next step, TMA acts as a catalyst, and the Al atoms in Al-CH_3_ initiate the interaction with TPS and the polymerization reaction of silanol to form an Si–O linkage within the polymer.^48,70^ In this mechanism, the Al atoms from TMA could be selectively localized in a polymer by reacting with any active functional groups such as carbonyl (C=O), ester (C–O–R), and amine (−NH_2_), and the subsequent silanol polymerization should create siloxane (Si–O–Si) chains within the polymer to generate localized SiO_2_ as shown in Figure 6 and in the equations below.

The first step of the reaction for these four polymers with TMA is proposed in eq 1, where asterisks indicate species within the polymer, and all other molecules are in the vapor phase:
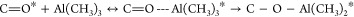
1

In eq 1, TMA reacts with carbonyl groups in the polymer to form a weakly bound intermediate complex. This complex can subsequently react to form a permanent covalent linkage to the polymer, as is predominantly the case for PCL and PBMA. Alternatively, the complex can dissociate during the subsequent TPS reaction to liberate the carbonyl, as seen for PMMA and PEMA.

Following the reaction of TMA with the polymer, we hypothesize that the subsequent TPS reaction occurs via the mechanism proposed for rapid SiO_2_ ALD using TMA and TPS:^48,70^
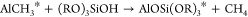
2
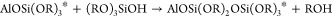
3

In eq 2, TPS reacts with AlCH_3_ in the polymer to form an Al–O–Si bond and liberate methane. In eq 2, additional TPS can insert at the Al catalytic center to create a siloxane polymer chain and liberate tert-pentanol (ROH). Subsequent cross-linking reactions occur that convert the siloxane chain to SiO_2_ and convert the Si–OR to Si–OH as described in refs (48) and (70). The cross-linking reactions densify SiO_2_ such that TPS diffusion to the Al catalytic center is restricted, and this terminates the rapid SiO_2_ process. The reactions described in eq 1–eq 3 are shown schematically in Figure 6. During additional SiO_2_ SIS cycles, TMA can react with the Si–OH groups, and this will restart the siloxane polymerization reactions shown in eq 2, eq 3 to deposit additional SiO_2_ in the polymer. However, the SiO_2_ SIS will ultimately terminate when the polymer densifies and the diffusion of TMA and TPS is restricted.

### 4.2 Insight into the Interactions of Different Polymers with the Precursors

The percentage of C=O groups participating in the SiO_2_ SIS can be calculated by comparing the integrated C=O loss peak and shifted C=O peak following the TMA reaction with the initial C=O intensity in the clean polymer. These values are small for PMMA (4%, Figure 2) and PEMA (16%, Figure 3). Similarly, we can calculate the percentage of C=O restored following the subsequent TPS exposure to be 37% for PMMA and ∼80% for PEMA. The values of the interacted and released C=O are summarized in Table 1. The C=O and C–O–R peaks in PMMA and PEMA show similar spectral shifting upon reaction with TMA and TPS indicating similar chemical interactions. This shifting and restoration of some of the C=O and C–O–R groups are due to weak complex formation C=O···Al(CH_3_)_3_ after TMA dose and dissociation of some of these weakly bound complexes after TPS dose, respectively. The weak complex formation is mostly due to the physisorption of TMA in the polymer, and the restoration of some of the C=O and C–O–R groups is due to the desorption of these weakly bound species over time (purge time) which is similar to the Al_2_O_3_ SIS precursor interactions with PMMA as discussed in our previous work.^54^ The desorption of the weakly bound species for PMMA-TMA interaction was evident from our previous work.^55^ To evaluate the stability of the PEMA-TMA complexes, we performed a purge time study as shown in Figure S5 after the first TMA dose on PEMA. We observed a loss of the interacted C=O and C–O–R spectral features versus time, indicating a weak interaction with TMA as seen previously for PMMA.

**Table 1:** Percentage of C=O Group Interactions after First TMA Exposure and the Percentage of Released C=O Groups after TPS Exposure

polymer name	interacted C=O after TMA exposure (%)	released C=O after TPS exposure (%)
PMMA	4	37
PEMA	16	80
PCL	99	0
PBMA	88	0

In contrast to PMMA and PEMA, almost all of the C=O and C–O–R groups participated in the SIS reaction with TMA for PCL and PBMA. This can be seen clearly in Figure 4 spectrum 4b.1 (99% C=O loss for PCL) and Figure 5 spectrum 5b.1 (88% C=O loss for PBMA). Even though we observe spectral shifting for C–O–R group in both PCL and PBMA, and for C=O in PBMA, these peaks do not reappear after the TPS dose indicating these groups from PCL and PBMA participated in an irreversible reaction with TMA followed by TPS.

The interactions of PCL and PBMA with TMA and TPS can be explained by the following steps: (i) absorption of TMA in the bulk of polymer films, (ii) intermediate C=O···Al(CH_3_)_3_ complex formation (which contributes to the spectral shifting), (iii) formation of a stable covalent bond of C–O–Al(CH_3_)_2_, and (iv) C–O–Al(CH_2_)_2_–Si–O after TPS exposure, as explained in eq 1-eq 3. The interacted C=O group in the PCL polymer does not show any spectral shift in the FTIR spectrum (spectrum 4a.1/S3a.1), indicating stable and irreversible compound formation, possibly from the beginning throughout the bulk of the polymer. In our previous work, a similar trend was observed for the interaction between PCL and Al_2_O_3_ precursors TMA-H_2_O.^56^

### 4.3 Reasons for Variation in Interactions for Different Polymers

We have contemplated the reasons for the variation in interaction for these polymers with TMA and the factors we have considered based on the observation from this work and our previous work on Al_2_O_3_ SIS in PMMA and PCL^56^ are: (i) the positioning of the C=O and C–O–R groups in the polymers, in the backbone structure or in the side chain, and (ii) the glass transition temperature and melting point of the polymers.

From Figure 1, we can see that for PMMA, PEMA, and PBMA, the C=O group position is in the side chain of the polymer and, for PCL only, it is in the backbone. As mentioned before, PMMA and PEMA show weakly bound reversible complex C–O···Al(CH_3_)_2_ formation, whereas PCL and PBMA show stable C=O–Al(CH_3_)_2_ formation, which clearly indicates that the reaction dynamics of these polymers with TMA followed by TPS are not correlated to the positioning of the C=O group. In our previous study of Al_2_O_3_ SIS in PMMA and PCL, the role of C=O positioning was inconclusive as the C=O position is in the backbone structure for PCL and in the side chain for PMMA.

**Figure 7:**
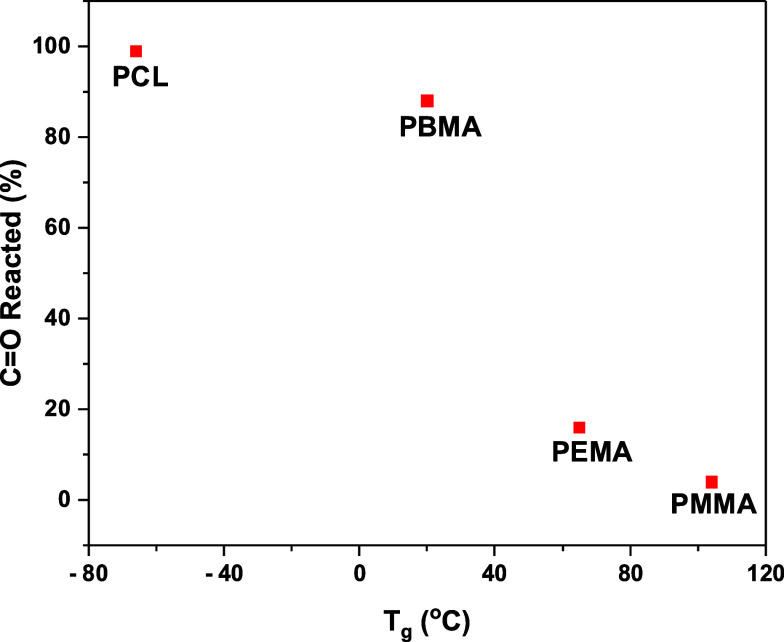
Plot of the percentage of CO reacted from FTIR measurements versus *T*_g_ for the four polymers in this study.

Next, we considered the glass transition temperature (*T*_g_) and melting point (*T*_m_) of these polymers, and we summarize the available *T*_g_ and *T*_m_ data in Table 2. The values in Table 2 and in Figure 7 show that we can categorize PMMA (*T*_g_ = 104 °C and *T*_m_ = 165 °C) and PEMA (*T*_g_ = 65 °C and *T*_m_ = 120 °C) as high Tg and *T*_m_ polymers, whereas PCL (*T*_g_ = −66 °C, and *T*_m_ = 60 °C) and PBMA (*T*_g_ = 20 °C, and *T*_m_ = not found) as the lower *T*_g_ and *T*_m_ polymers. The higher *T*_g_/*T*_m_ PMMA and PEMA show partial and reversible interaction of C=O with TMA, whereas the lower *T*_g_/*T*_m_ PCL and PBMA show complete and irreversible interaction of C=O with TMA. From the data shown in this work, it is evident that the thermal characteristics of the polymers play a crucial role, and most likely, the lower *T*_g_ and *T*_m_ of PCL and PBMA compared to PMMA and PEMA facilitated faster precursor diffusion and enabled the PCL and PBMA to assume a lower energy configuration around the precursor molecules that stabilize the polymer–precursor bonding. Figure 7 plots the percentage of CO reacted from the in situ FTIR measurements versus *T*_g_ for the four polymers in this study and demonstrates that the extent of the C=O reaction by the TMA is greater for the lower *T*_g_ polymers.

**Table 2:** Glass Transition Temperatures (*T*_g_)^71,72^ and Melting Temperatures (*T*_m_) of the Polymers in this Study^73−75^

**polymer name**	**glass transition temperature (***T*_**g**_**) in °C**	**melting temperature (***T*_**m**_**) in °C**
PMMA	104	165
PEMA	65	120
PCL	–66	60
PBMA	20	not available

### 4.4 Relative Extent and Nature of Si–O Infiltration in the Polymers

**Figure 8:**
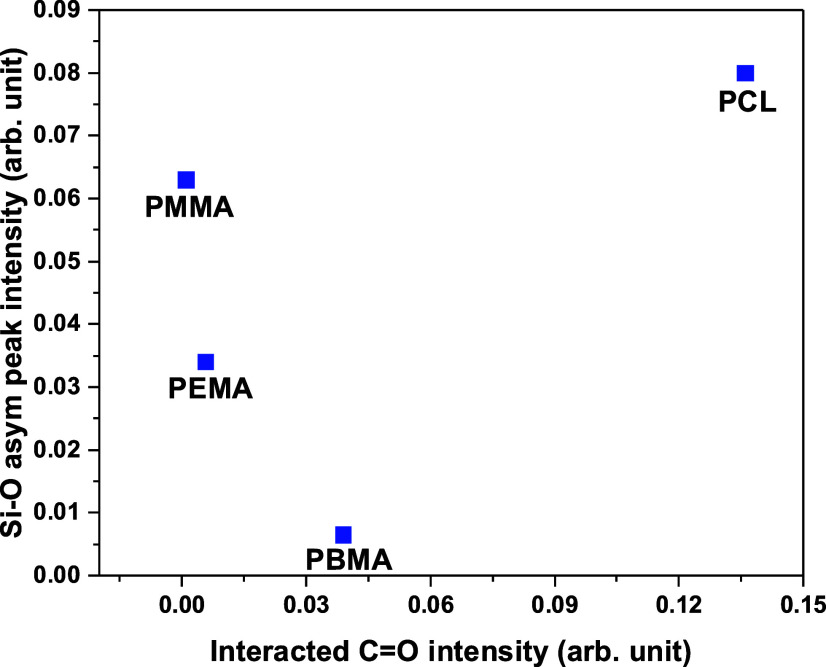
Magnitude of the Si–O asymmetric peak versus magnitude of the C=O loss peak after three SIS cycles (spectra 2b.1, 3b.1, 4b.1, and 5b.1) showing relative extent of SiO_2_ SIS in the polymers.

To understand and quantify the correlation between polymer functional group interaction and the extent of SiO_2_ infiltration in the polymer, the relative peak intensity changes for the major polymer functional group (C=O) and the deposited SiO_2_ material (Si–O species) from the FTIR difference spectra after the completion of three cycles of SIS are compared for the four polymers. Figure 8 shows the plot of interacting C=O and deposited Si–O species (asymmetric vibration mode) from the FTIR spectra. It also provides information about the relative extent of the infiltration of the polymer-SiO_2_ material after three SIS cycles. The extent of this interaction provides a good understanding of the material formation throughout the polymers. From Figure 8, it is evident that PCL shows the greatest participation of C=O groups in the interaction with SiO_2_ precursors, and the higher intensity of the Si–O asymmetric mode also indicates the relatively higher growth of SiO_2_ in PCL compared to the other polymers. Note that the C=O groups in PCL participate in an irreversible reaction with TMA; hence, we do not see the return of the C=O peak after TPS interaction as shown in spectrum 4a.2. Besides PCL, the other three polymers PMMA, PEMA, and PBMA show variations in C=O participation versus Si–O formation. Among these three polymers, PBMA shows the maximum participation of C=O, followed by PEMA and PMMA; however, the amount of Si–O asymmetric peak formation is not in the same order. Note that PCL and PBMA also show noticeable Si–O symmetric vibration peaks in the corresponding spectra 4b.1 and 5b.1, respectively. The PBMA interaction is interesting as all the available C=O participated in the reaction; however, the Si–O peak intensity is not significant (neither is the Si–O symmetric peak, which is not plotted in Figure 8), which might be due to the formation of intermediate complexes, as shown in eq 2, but lack of polymerization reaction and subsequent siloxane formation, as shown in eq 3. On the contrary, for PMMA less C=O groups participated in the interaction; however, we see an intense Si–O peak indicating that most of the Si–O formation probably happened from subsequent silanol diffusion and polymerization reaction. We also note that the Si–O symmetric peak was mostly visible only after the first SIS cycle for all four polymers, and from the second cycle onward, the intensity of the Si–O asymmetric stretching peak increased compared to Si–O symmetric stretching peak. The Si–O asymmetric and symmetric stretching peaks are due to Si–O formation with different bond lengths and bond energies within the polymer network. The appearance of the dominant Si–O asymmetric peak might be due to the majority of the reaction and formation of Si–O on existing SiO_2_ nuclei embedded in the polymer during the second and third SiO_2_ SIS cycles instead of the reaction with the polymer.

## 5 Conclusions

The in situ FTIR results shown in this work provide insight into the TMA-catalyzed SiO_2_ SIS process in four different polymers for applications in hybrid material synthesis and nanopatterning. We observed variations in functional group interactions for these four polymers with the two precursors even though they share the same C=O and C–O–R functional groups. PCL and PBMA show a stable interaction of all the functional groups with TMA compared to PMMA and PEMA, where we observed a fraction of the functional group participating in the interaction, and the reaction was reversible. We interpreted this variation in the reaction as most likely due to the lower *T*_g_ and *T*_m_ of PCL and PBMA compared to PMMA and PEMA, which enables faster precursor diffusion and stable polymer-precursor bonding. After the TPS dose, SiO_2_ forms in these polymers, as observed by FTIR. However, the Si–O intensity is not directly correlated with the amount of functional group interactions of these polymers. The PCL polymer shows maximum Si–O formation, followed by PMMA, PEMA, and PBMA. Even though all the functional groups of PBMA interacted after TMA exposure similar to PCL, we interpreted that the lower intensity of the Si–O peaks might result from intermediate complex formation due to incomplete silanol transformation to siloxane. The insight gained from the FTIR results in this work will be significant to establishing and optimizing the fabrication of SiO_2_ nanopatterns. In the future, we plan to fabricate and optimize SiO_2_ nanostructured patterns using the BCP nanostructures of these polymers as a template and using TMA-TPS as the SIS precursors.

## Supporting Information Available

FTIR difference spectra are shown for each precursor exposure on PMMA during three SIS cycles separately, FTIR difference spectra are shown for each precursor exposure on PEMA during three SIS cycles separately, FTIR difference spectra are shown for each precursor exposure on PCL during three SIS cycles separately, FTIR difference spectra are shown for each precursor exposure on PBMA during three SIS cycles separately, and FTIR difference spectra are shown for PEMA polymer after first TMA exposure and during purge time after TMA exposure (PDF)
