# Supplementary material for: Sequential Infiltration Synthesis of Silicon Dioxide in Polymers with Ester Groups—Insight from In Situ Infrared Spectroscopy
Source: J Phys Chem C Nanomater Interfaces. 2024 Apr 3;128(15):6346–56. doi: 10.1021/acs.jpcc.3c07571 (PMC11033938; doi:10.1021/acs.jpcc.3c07571)
Supplement: Supplementary file 1 — jp3c07571_si_001.pdf [file jp3c07571_si_001.pdf]

Supplemental Information for:

Sequential Infiltration Synthesis of Silicon Dioxide in Polymers with Ester Groups– Insight from In Situ Infrared Spectroscopy

Mahua Biswas[a], Vepa Rozyyev[b,c,d], Anil U. Mane[b,c], Amelia Korveziroska[a], Uttam Manna[a], Jeffrey W. Elam[b,c*]*

[a]Department of Physics, Illinois State University, Normal, Illinois, 61704, United States

[b]Applied Materials Division, and [c]Advanced Materials for Energy-Water Systems (AMEWS) Energy Frontier Research Center, Argonne National Laboratory, 9700 South Cass Avenue, Lemont, Illinois, 60439, USA, [d]Pritzker School of Molecular Engineering, The University of Chicago, Chicago, Illinois, 60637, United States

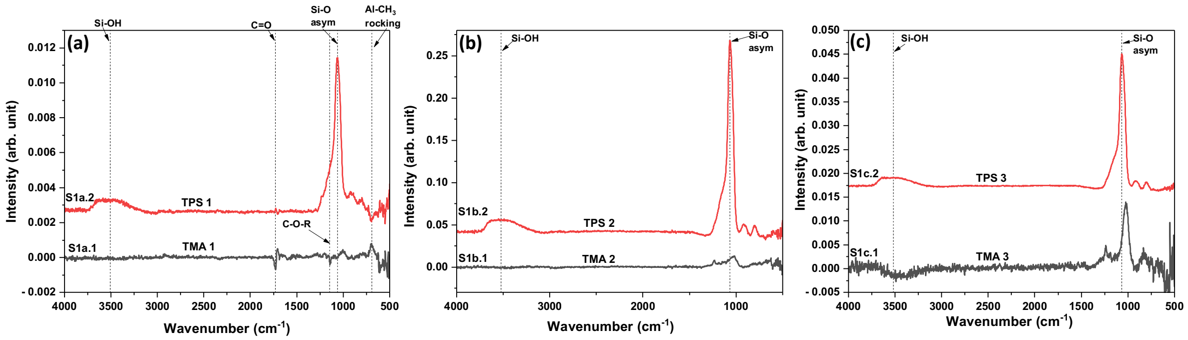

Figure S1: FTIR difference spectra recorded after each precursor exposure on PMMA during three SIS cycles, (a) after first cycle, (b) after second cycle, and (c) after third cycle. S1a.1, S1b.1, and S1c.1 are the difference spectra after the TMA exposures and S1a.2, S1b.2, and S1c.1 are the difference spectra after the TPS exposures.

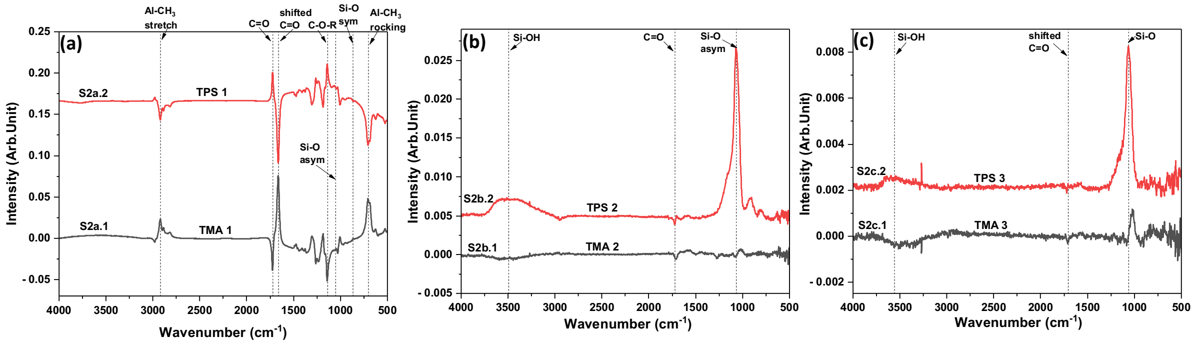

Figure S2: FTIR difference spectra recorded after each precursor exposure on PEMA during three SIS cycles, (a) after first cycle, (b) after second cycle, and (c) after third cycle. S2a.1, S2b.1, and S2c.1 are the difference spectra after the TMA exposures and S2a.2, S2b.2, and S2c.1 are the difference spectra after the TPS exposures.

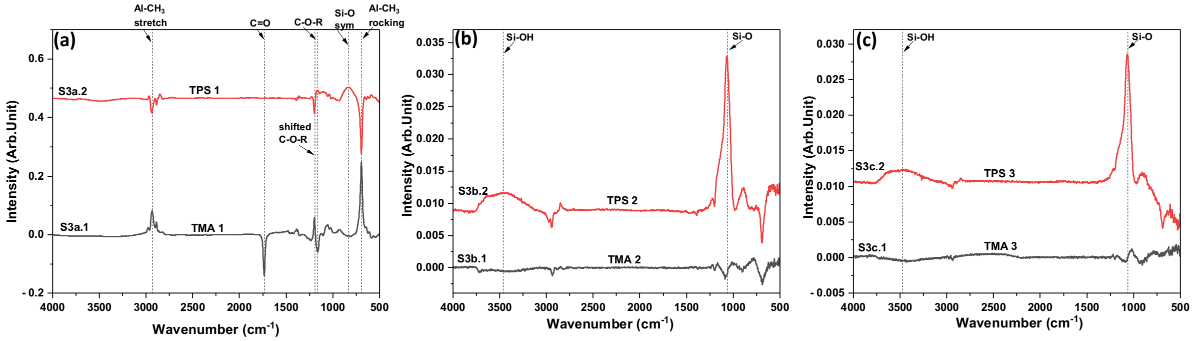

Figure S3: FTIR difference spectra recorded after each precursor exposure on PCL during three SIS cycles, (a) after first cycle, (b) after second cycle, and (c) after third cycle. S3a.1, S3b.1, and S3c.1 are the difference spectra after the TMA exposures and S3a.2, S3b.2, and S3c.1 are the difference spectra after the TPS exposures.

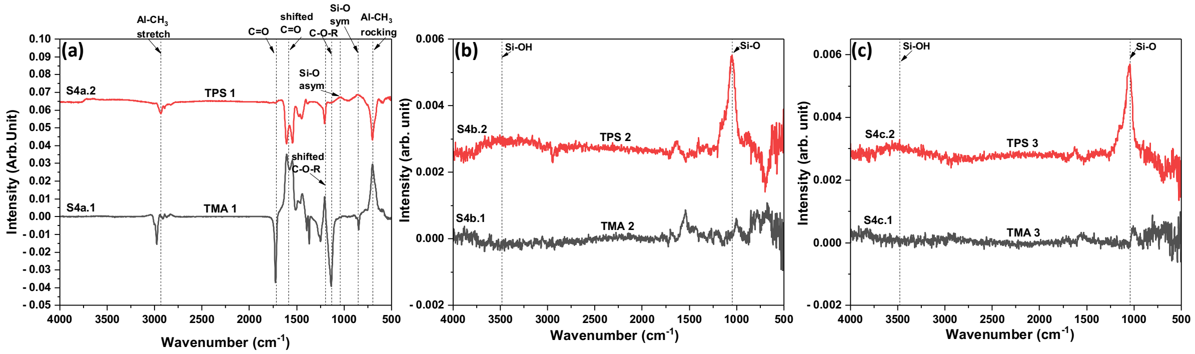

Figure S4: FTIR difference spectra recorded after each precursor exposure on PBMA during three SIS cycles, (a) after first cycle, (b) after second cycle, and (c) after third cycle. S4a.1, S4b.1, and S4c.1 are the difference spectra after the TMA exposures and S4a.2, S4b.2, and S4c.1 are the difference spectra after the TPS exposures.

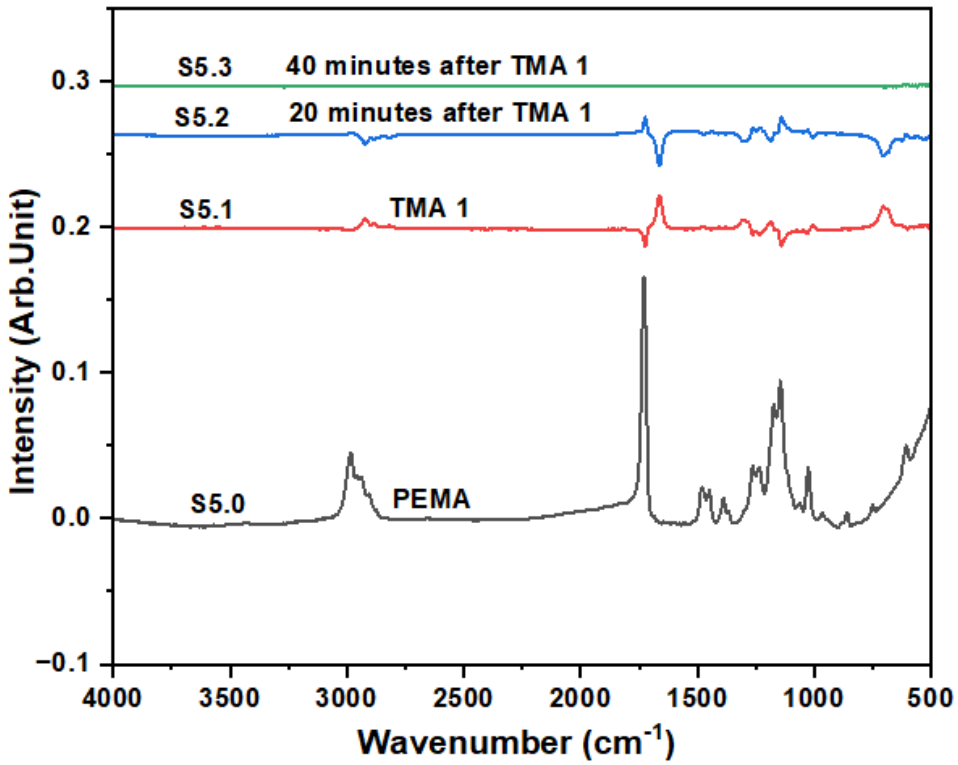

Figure S5: FTIR difference spectra recorded after first TMA exposure and during purge time after TMA exposure. S5.0 is grown PEMA spectrum, S5.1difference spectra after first TMA exposure, S5.2 and S5.3 are recorded after 20 minutes and 40 minutes, respectively following first TMA exposure to study the stability of the complexes formed during purge time.
